# Mutations in the acetolactate synthase (ALS) enzyme affect shattercane (*Sorghum bicolor*) response to ALS-inhibiting herbicides

**DOI:** 10.1186/s41065-023-00291-y

**Published:** 2023-06-21

**Authors:** Ismail M. Dweikat, Malleswari Gelli, Mark Bernards, Alex Martin, Amit Jhala

**Affiliations:** 1 Department of Agronomy and Horticulture, University of Nebraska, Lincoln, NE 68583 USA; 2 Department of Agronomy, Crop Science and Weed Control, Western Illinois University, Macomb, IL 61455 USA

**Keywords:** Amplification, Biotype, dose response, resistance level, sequencing, weed management

## Abstract

**Background:**

Shattercane [Sorghum bicolor (L.) Moench ssp. Arundinaceum (Desv.)] is a competitive weed in North America's corn, soybean, sorghum, and other agronomic crops. Control of shattercane with POST herbicides in corn became possible with the introduction of acetolactate synthase (ALS)-inhibiting herbicides in the 1980s, and their extensive use resulted in the evolution of ALS-inhibitors resistant shattercane.

**Results:**

Shattercane seeds were collected from 16 south-eastern and south-central Nebraska fields that were treated with primisulfuron for three consecutive years. Three resistant plants were found in greenhouse evaluations of more than 30,000 plants. Results from a greenhouse bioassay conducted to assess the response of each shattercane biotype to ALS-inhibiting herbicides showed a differential response to ALS inhibitors within and between chemical classes. Biotype P8-30 was resistant or partially resistant to all ALS-inhibiting herbicides applied and displayed a unique amino acid sequence substitution (Trp574 to Leu) relative to the other two resistant biotypes, P2-205 and P9-102. Whole plant dose–response studies confirmed a 4- to the 12-fold level of primisulfuron resistance in three shattercane biotypes compared with the known primisulfuron-susceptible shattercane biotype. The ALS gene was sequenced using primers designed from the corn ALS sequence to identify mutations in the ALS gene that confer resistance. A total of seven nucleotide substitutions were detected in the three herbicide-resistant biotypes P2-205, P8-30, and P9-102. These biotypes are being crossed to adapted sorghum lines (grain, sweet, and forage) to broaden germplasm with resistance to ALS-inhibiting herbicides.

**Conclusion:**

The discovery of these mutants should accelerate the development of sorghum genotypes that tolerate ALS-based herbicides, which provide additional choices for sorghum farmers to control weeds, especially grasses, in their fields.

## Background

Acetolactate synthase (*ALS*) is a nuclear gene that encodes acetolactate synthase (ALS; EC 4.1.3.18), a key enzyme involved in biosynthesis of the branched-chain amino acids valine, leucine, and isoleucine [1] essential for plant growth and development [2,and inhibition of their synthesis is lethal to many plant species The enzyme is the inhibition target of more 50 commercial herbicides covering five structurally different classes of chemicals [3], including sulfonylurea (SU), imidazolinone (IMI), pyrimidinylthiobenzoate (PTB), triazolopyrimidine sulfonanilide (TPS), and sulfonylamino carbonyl triazolinones (SCT) [4].

ALS-inhibiting herbicides have been widely used in agriculture and currently comprise the largest site-of-action group for weed management, primarily in agronomic crops. They control numerous weed species at low use rates, provide weed control in multiple crops, and possess low mammalian toxicity [2, 5–8]. The herbicides in this group display a specific site of action, leading to selection for resistance with intensive repeated applications [9]. Weeds have evolved resistance to 21 of the 31 known herbicide sites of action [3]. The ALS-inhibiting herbicide group has the most significant number of resistant weeds (167) [3].

Spontaneous mutations confer decreased ALS sensitivity at the *ALS* locus ([10]. Single amino-acid substitution at one of seven different positions in the *ALS* gene confers herbicide resistance [11, 12]. The substitutions, Asp_376_ [13], Ala_122_, Pro_197_, Ala_205_, Trp_574_, Ser_653_ [14], and Gly_654_ [15], produce distinct patterns of resistance to different ALS inhibiting herbicides. Substitution of Pro_197_ results in resistance to SU and TPS herbicide classes [16], while Ala_122_ and Ala_205_ enable resistance to IMI, Ser_653_ confers resistance to IMI and PTB classes, Gly_654_ confers resistance to SU, IMI, and SCT classes, and Trp_574_ and Asp_376_ provide broad resistance to all five classes of ALS inhibiting herbicides [17, 18]. Pro197 to Ser was the most frequent substitution among these substitutions observed in weed biotypes [17].

Sorghum is a major cereal crop in the semi-arid tropics of the world, with tolerance to several biotic and abiotic stresses and the ability to grow in water-limited environments [19]. Consequently, sorghum is a candidate alternative grain crop for ethanol and feed, particularly in geographic areas susceptible to dry soil conditions [20]. A Few pre-emergence (PRE) herbicides are labeled in sorghum that provides broad-spectrum residual control of weeds; however, achieving post-emergence POST) control of grass weeds represents a significant management challenge that must be addressed for the crop to be economically viable.

Over the past decade, researchers at Kansas State University developed a grain sorghum cultivar named INZEN that is resistant to several ALS-inhibiting herbicides by transferring a resistance gene from shattercane. This cultivar results from wild genes expressed in cultivated sorghum through genetic recombination or from introgression of cultivated sorghum with johnsongrass [*Sorghum Halepense* (L.) Pers] [21–23]. Due to the repeated use of ALS inhibitors, Threehattercane biotypes have evolved resistance to ALS-inhibiting herbicides in sorghum production fields in southeastern and south-central Nebraska in the late 1990s. Due to growers' complaints regarding shattercane control failure, shattercane seeds were collected for experimentation. This study aimed to understand the resistance mechanism to ALS-inhibiting herbicides in these shattercane biotypes. Greenhouse experiments were conducted to evaluate the response of selected ALS-inhibitors shattercane biotypes and a susceptible wild-type biotype to the four classes of ALS-inhibiting herbicides: SU, PTB, TP, and SCT. The objectives of this research were to confirm putative ALS inhibiting herbicide resistance in shattercane biotypes via a dose–response study and to identify DNA polymorphisms in ALS genes from the mutant lines, enabling the identification of novel resistance alleles.

## Results and discussion

### Assay for resistance

The three mutant shattercane biotypes showed differential responses to various ALS-inhibiting herbicides (Table 1). Resistance levels of mutants were determined based on the relative % of dry mass produced (Table 2) and visible symptoms observed at 21 days after herbicide treatment (Table 3) compared to the wild type. Biotype P2-205 conferred complete resistance to Propoxycarbazone and Chlorsulfuron, producing a very high relative % dry mass compared to wild type (Table 2) and visual symptoms ranged from 0 to 5, indicating no observed injury in two runs (Table 3). This biotype showed partial resistance to imazamox, foramsulfuron, primisulfuron, and thiencarbazone, maintaining minimal growth after the treatment (visual symptoms ranged from 40 to 80). The nucleotide sequence of ALS for this biotype showed three point mutations encoding Ala30 to Gly, Pro185 to Leu, and Glu634 relative to the susceptible biotype. These amino acid substitutions conferred resistance to SU and triazolone herbicides.

**Table 1:** Sequence substitutions in the acetolactate synthase (ALS) gene and resistance to herbicide active ingredient of three ALS inhibitors-resistant shattercane biotypes relative to the known susceptible biotype. Resistance was defined as having a lower percentage dry matter reduction when treated with herbicide compared to the wild type. Penoxsulam, the one herbicide in the triazolopyrimidine chemical family that claimed control of some grass species on its label, did not reduce the growth of any shattercane mutant biotype

	Sequence substitutions		
Position	P2-205	P9-102	P8-30
91	Ala_30_-Gly	Ala_30_-Gly	Ala_30_-Gly
554	Pro_197_-Leu	Pro_197_-Leu	
1685			Trp_574_ -Leu
1899	Glu646Asp		
1642			
	Resistance to herbicide active ingredient		
Chemical family	P2-205	P9-102	P8-30
Imizadolinones	Imazamox^a^		Imazamox
			Imazaquin
			Imazethapyr
Pyrimidinyloxybenzoic acids			Bispyribac^a^
Sulfonylureas	Chlorsulfuron	Chlorsulfuron	Chlorsulfuron
	Foramsulfuron^a^	Nicosulfuron	Foramsulfuron
	Primisulfuron^a^	Primisulfuron^a^	Nicosulfuron
			Primisulfuron
			Rimsulfuron^a^
Triazolones	Propoxycarbazone	Propoxycarbazone	Propoxycarbazone
	Thiencarbazone^a^	Thiencarbazone^a^	Thiencarbazone

^a^Partial resistance

**Table 2:** Shattercane dry weight as a percent of the nontreated control in response to ALS-inhibiting herbicides applied at labeled rates. Low numbers indicate greater reduction in growth. The experimental run x herbicide x population and the herbicide x population interactions were significant (*p* = 0.001), so data were analyzed by herbicide for each run. Each value represents the mean of four replications. The wild type is a susceptible sorghum line (Ck 60). Means in a row followed by the same letter are not statistically different, *p* = 0.05

Herbicide	Run	P2-205		P9-102		P8-30		Wild type	
Imazamox	1	7	b	4	b	147	a	3	b
Imazamox	2	10	b	13	b	85	a	4	b
Imazaquin	1	10	b	8	b	152	a	11	b
Imazaquin	2	10	c	22	b	88	a	8	c
Imazethapyr	1	7	b	6	b	112	a	3	b
Imazethapyr	2	11	b	16	b	97	a	6	b
Bispyribac	1	4	b	4	b	41	a	3	b
Bispyribac	2	8	bc	13	b	30	a	4	c
Chlorsulfuron	1	78	b	52	c	109	a	11	d
Chlorsulfuron	2	95	a	71	b	98	a	49	c
Foramsulfuron	1	16	b	99	a	118	a	4	b
Foramsulfuron	2	14	b	90	a	112	a	4	b
Nicosulfuron	1	5	b	103	a	88	a	4	b
Nicosulfuron	2	7	b	105	a	118	a	6	b
Primisulfuron	1	13	b	19	b	125	a	4	b
Primisulfuron	2	15	b	27	b	113	a	5	b
Rimsulfuron	1	5	b	4	b	19	a	3	b
Rimsulfuron	2	7	ab	11	ab	13	a	4	b
Propoxycarbazone	1	87	a	86	a	106	a	16	b
Propoxycarbazone	2	103	a	102	a	68	b	26	c
Thiencarbazone	1	10	b	6	b	139	a	3	b
Thiencarbazone	2	10	bc	18	b	118	a	2	c
Penoxsulam	1	86	a	86	a	98	a	108	a
Penoxsulam	2	106	ab	74	ab	102	ab	119	a
Glyphosate	1	3	b	3	b	5	a	3	b
Glyphosate	2	4	ab	5	a	2	d	3	cd

Values highlighted in yellow represent resistance to the herbicide mode of action. Values in gray represent partial resistance to the herbicide mode of action

**Table 3:** Shattercane injury estimated visually 21 d after treatment (DAT) in response to ALS-inhibiting herbicides applied at labeled rates. High numbers indicate greater reduction in growth, discoloration, or growth deformation. The Run x herbicide x population and the herbicide x population interactions were significant (*p* = 0.001), so data were analyzed analyzed by herbicide for each run. Each value represents the mean of four replications. The wild type is a susceptible sorghum line Ck60

Herbicide	Run	P2-205		P9-102		P8-30		Wild type	
Imazamox	1	**75**	c	98	b	0^a^	d	100	a
Imazamox	2	**70**	d	80	c	0^a^	e	100	a
Imazaquin	1	60	b	70	a	0^a^	c	60	b
Imazaquin	2	63	ab	55	b	0^a^	c	71	a
Imazethapyr	1	71	c	93	b	0^a^	d	100	a
Imazethapyr	2	70	b	74	b	0^a^	c	92	a
Bispyribac	1	100	a	98	a	**32**	b	100	a
Bispyribac	2	77	c	65	d	**30**	e	99	a
Chlorsulfuron	1	0^a^	d	10^a^	c	0^a^	d	65	b
Chlorsulfuron	2	0^a^	c	0^a^	c	0^a^	c	23	b
Foramsulfuron	1	**46**	b	0^a^	c	0^a^	c	95	a
^a^Foramsulfuron	2	**45**	c	0^a^	d	0^a^	d	93	a
Nicosulfuron	1	99	a	0^a^	b	0^a^	b	99	a
Nicosulfuron	2	93	b	0^a^	c	0^a^	c	97	a
Primisulfuron	1	**55**	b	**48**	c	3^a^	d	100	a
Primisulfuron	2	**48**	b	**45**	b	0^a^	c	100	a
Rimsulfuron	1	100	a	100	a	**50**	b	100	a
Rimsulfuron	2	85	b	91	ab	**58**	c	100	a
Propoxycarbazone	1	5^a^	b	0^a^	b	3^a^	b	58	a
Propoxycarbazone	2	0^a^	c	0*	c	0^a^	c	30	b
Thiencarbazone	1	**66**	b	**70**	b	0^a^	c	100	a
Thiencarbazone	2	**70**	c	**60**	d	0^a^	e	100	a
Penoxsulam	1	3	a	0	a	1	a	0	a
Penoxsulam	2	0	a	0	a	0	a	2	a
Glyphosate	1	100	a	100	a	100	a	100	a
Glyphosate	2	100	a	100	a	100	a	100	a

Highlighted values represent partial resistance relative to the wild type in both runs of the experiment

^a^represent resistance to the herbicide mode of action

Biotype P9-102 shared two mutations in common with P2-205 but displayed a different spectrum of resistance. The relative percentage of dry mass produced by P9-102 following treatment with nicosulfuron was higher (Table 2) than P2-205 and P8-30. This genotype also had a higher drymass than the wild type when treated with chlorsulfuron and propoxycarbazone. Biotype P9-102 showed partial resistance to primisulfuron and thiencarbazone (Table 1). Visual symptoms were lower (0 to 10) at 21 days after treatment, indicating that P9-102 was resistant to SU and triazolones. The only herbicide that did not reduce the dry matter of P2-205 and P9-102 was Penoxsulam, belonging to the triazolopyrimidine class. Herbicides from this class have limited activity on grass species. Penoxsulam was selected to represent this chemical family because it controls some grass weeds (Echinochloa sp).

Biotype P8-30 exhibited enhanced resistance relative to wild-type to all ALS-inhibiting herbicides tested (Table 1). A higher percentage of relative dry mass produced after herbicide treatment indicated cross-resistance to all ALS inhibitors (Table 2). For bispyribac and rimsulfuron, biotype P8-30 showed partial resistance, and growth was reduced approximately 65% and 84%, respectively. Visual ratings allowed distingushment between plants that were severely stunted but still capable of completing their life cycle (partial resistance) and plants that were severely stunted and dying (susceptible).

Post-emergence herbicide treatment options for controlling grasses are limited in grain sorghum. ALS-inhibiting herbicides effectively control many grass species in corn, but these herbicides are not an option in conventional sorghum due to sorghum’s susceptibility. The successful identification of ALS-resistant grain sorghum could enable the use of ALS-inhibiting herbicides for POST weed control.

### Dose–response study

Treatment-by-experiment interaction was not significant for the dose–response study of primisulfuron; therefore, data were pooled over experiments. A test of lack of fit at a 95% significance level was not significant for any of the curves tested, providing evidence that the models were sufficiently fit (data not shown) [24]. The labeled rate of primisulfuron (40 g ai ha^–1^) resulted in ≥ 90% control of the known susceptible shattercane biotype compared to ≤ 15% control of the putative primisulfuron resistant biotypes 28 DAT (days after treatment) (data not shown), confirming resistance (Table 4). The putative primisulfuron resistant shattercane biotypes showed a 4 to 12-fold level of resistance to primisulfuron relative to the known primisulfuron susceptible shattercane biotype, depending on the biotype being investigated (Table 4).

**Table 4:** Regression parameter estimates and effective primisulfuron doses in greenhouse dose–response studies resulting in 50% (ED_50_) and 90% (ED_90_) control of primisulfuron-susceptible and resistant shattercane biotypes

Shattercane biotype^a^	Primisulfuron					Resistance level^c^
	Parameter estimates^b^			Effective doses^b^		
	B (± SE)^a^	C (± SE)^a^	D (± SE)^a^	ED_50_ (± SE)^a^	ED_90_ (± SE)^a^	
				g ai ha^─1^		
Primisulfuron-susceptible	-1.5 (0.14)	0.09 (0.03)	98.9 (1.8)	18 (4)	32 (7)	-
Primisulfuron-resistant (P9-102)	-1.1 (0.1)	-0.12 (0.03)	108 (9)	74 (18)	128 (25)	4 ×
Primisulfuron-resistant (P2-205)	-1.3 (0.2)	-0.14 (0.04)	105 (6)	102 (21)	192 (29)	6 ×
Primisulfuron-resistant (P8-30)	-1.4 (0.2)	-0.17 (0.02)	112 (10)	201 (45)	384 (52)	12 ×

^a^*Abbreviations*: *DAT* Days after treatment, *ED* Effective dose, *SE* Standard error

^b^Regression parameters B, C and D represent slope, lower and upper-limit of the four-parameter log-logistic model, respectively, and were determined by using the nonlinear least-square function of the statistical software R. ED_50_, effective primisulfuron dose required for 50% control of shattercane at 28 DAT; ED_90_, effective primisulfuron dose required for 90% control of shattercane at 28 DAT

^c^Resistance level was calculated by dividing the ED_90_ value of the primisulfuron-resistant shattercane biotype by that of the primisulfuron-susceptible biotype

### Sequencing of the *ALS* Gene

The *ALS* gene, spanning a length of 2270 bp, was completely sequenced from sorghum genotype CK60, wild-type shattercane, and the three shattercane mutants P2-205, P9-102, and P8-30, and nucleotide sequence comparisons showed that ALS gene sequences from the three mutants were highly conserved relative to wild type. A nucleotide substitution of GCC to GGC at position 91 was observed in all three mutants and coded for an Ala_30_ to Gly substitution. Another nucleotide substitution of CCG to CTG at position 554 was observed in mutants P2-205 and P9-102 for a Pro_197_ to Leu substitution. The substitutions at this proline have been reported in previous studies [25–27] to confer high-level resistance to sulphonylurea (SU) herbicides with little or no resistance to IMI herbicides. A nucleotide substitution of GAG to GAT at position 1899 (based on the Arabidopsis ALS amino acid sequence) was also observed in mutant P2-205, where aspartate at this position appears to be highly conserved among all known wild-type enzymes [28]. Falco et al. [29] reported that substituting glutamate for this aspartate confers resistance to SU in yeast following selection. The nucleotide substitution of TGG to TTG at position 1685 was observed in mutant P8-30, encoding Trp_574_ to Leu, a substitution that has not been previously reported. In total, 17 amino acid substitutions that confer herbicide resistance have been reported in plants, yeast, bacteria, and green algae or natural field-selected biotypes [30], providing background for assessing mutation outcomes.

The three selected shattercane mutants exhibited average growth relative to the susceptible biotype. However, Rajcan et al. [31] showed that several Powell amaranth [*Amaranthus powellii* (S.) Wats.] biotypes with an amino acid substitution of Trp_574_ to Leu exhibited slow development, reduced biomass and leaf area, and distorted leaves relative to ALS inhibitor-susceptible biotypes. Hernandez et al. [32] also reported similar results in Johnsongrass. The Trp_574_ to Leu substitution confers resistance to tribenuron-methyl in catch weed bedstraw [33].

Likewise, substitution at Ala_122_ in ALS of common cocklebur (*Xanthium strumarium* L.) confers resistance to IMI herbicides [34]. An identical substitution was reported in the commercial field corn hybrid, ICI 8532 IT, and in sugar beet [*Beta vulgaris* (L.) line Sur] that also confer resistance to IMI herbicides [33, 35]. Substitution at Pro_197_ confers high-level resistance to SU herbicides with little or no resistance to IMI herbicides [25–27], whereas substitution at Ser_653_ confers high-level resistance to IMI herbicides with low resistance to SU herbicides (][36]. Ala_205_ and Trp_574_ substitutions have conferred cross-resistance to SU, IMI, PTB, and TP chemistries [33, 37]. Traditional breeding has been used to develop grain sorghum germplasm tolerant to acetolactate synthase (ALS)-inhibiting herbicides (Inzen Technology, DuPont). Inzen sorghum carries a double mutation in the ALS gene (Val 560 Ile and Trp 574 Leu) [38]. Variable patterns of resistance to ALS-inhibiting herbicide chemistries indicate that a resistance assessment is necessary for each ALS inhibitor-resistant weed biotype.

## Materials and methods

### Plant materials

Shattercane seeds were collected in 1990 years from fields from five locations in Nebraska previously treated with primisulfuron for three consecutive years. The total number of seeds were added to about 30,000 that produced 27,000 seedlings. All seedlings were evaluated under greenhouse conditions with 1x (40 g ai ha-1) and 0.25x (10 g ai ha-1) use rates of primisulfuron. Plants surviving the 1 × were treated a second time with a 2 × use rate (80 g ai ha-1) of primisulfuron. Plants surving the 0.25 × did not survive the higher rates and were discarded. The screening process resulted in the identification of three shattercane biotypes P2-205, P8-30, and P9-10, resistant to both 1 × and 2 × use rates of primisulfuron. All experiments included a susceptible (S) biotype of shattercane as a known primisulfuron-susceptible biotype.

### Assay for resistance

The bioassay experiment was conducted in a greenhouse as a randomized complete block design with five replications Seeds from three putative primisulfuron-resistant shattercane biotypes and one known primisulfuron susceptible biotype were planted in 0.9 L square plastic pots containing Miracle-Gro® Moisture Control® potting mix (The Scotts Company LLC, Marysville, OH). Plants were grown at 24 ± 2º C (day) and 19 ± 2º C (night) temperatures with a photoperiod of 16/8 light/dark with supplemental light provided by sodium halide lamps. Shattercane biotypes were thinned to 1 plant per pot at the 2-leaf stage. Herbicide treatments (Table 5) were applied when plants ranged from growth stage V3 to V5 and were 7 to 19 cm tall. At least one tiller had formed on most plants by the time of application. Herbicides were applied using an 8001 even flat fan nozzle at 207 kPa in asingle-tip track sprayer (DeVries Manufacturing, Hollandale, MN). The sprayer was calibrated to dilver 187 L ha-1 spray volume, and treatment solutions were prepared in distilled water. Visible plant injury ratings were recorded at 7, 14, and 21 days after treatment (DAT) on a scale of 0% to 100%, where 0% represents no injury and 100% represents plant death. At 21 DAT, plants were harvested at the soil surface, dried in a forced –air oven for 48 h at 70º C, and weighed to determine dry biomass. The experiment was conducted twice. Dry biomass data for each experimental unit were divided by the average dry matter of the non-treated control plants for that biotype and multiplied by 100 to determine the percent biomass relative to the nontreated control. Relative dry biomass estimates for all entries were calculated to determine growth in the three weeks following herbicide application. Data were subjected to ANOVA using Proc GLM of SAS 9.1 (SAS Institute Inc., Cary, North Carolina). The experimental x herbicide x biotypeinteraction was significant for each variable, so data for experimental runs were analyzed separately. The herbicide x population interaction was significant for each experimental run and each variable, so data were analyzed by herbicide to describe differences among the biotype Treatment means were separated using Duncan’s Multiple Range test.

**Table 5:** Acetolactate synthase (ALS) inhibiting herbicides and their use rates in a greenhouse bioassay to determine response of putative ALS inhibitors resistant and a known susceptible shattercane biotypes from Nebraska

Herbicide and adjuvant(s)	Rate	Unit	Chemical family
Chlorsulfuron	39.4	g ai/ha	Sulfonylurea
NIS	0.5	% v/v	
AMS	3360	g/ha	
Foramsulfuron	43	g ai/ha	Sulfonylurea
MSO	1.75	l/ha	
AMS	3360	g/ha	
Nicosulfuron	35.2	g ai/ha	Sulfonylurea
COC	1	% v/v	
AMS	2240	g/ha	
Primisulfuron	40	g ai/ha	Sulfonylurea
MSO	2.34	l/ha	
AMS	4500	g/ha	
Rimsulfuron	26.3	g ai/ha	Sulfonylurea
MSO	0.5	%v/v	
AMS	2240	g/ha	
Imazamox	52.5	g ai/ha	Imidazolinone
MSO	1	%v/v	
AMS	2240	g/ha	
Imazaquin	137	g ai/ha	Imidazolinone
COC	1.25	%v/v	
AMS	2240	g/ha	
Imazethapyr	70	g ai/ha	Imidazolinone
MSO	1	%v/v	
AMS	2240	g/ha	
Penoxsulam	49	g ai/ha	Triazolopyrimidine
COC	2.5	%v/v	
Bispyribac	37.5	g ai/ha	Pyrimidinyloxybenzoic acid
MSO	0.5	%v/v	
AMS	2240	g/ha	
Propoxycarbazone-sodium	44	g ai/ha	Triazolone
NIS	0.5	%v/v	
AMS	3360	g/ha	
Thiencarbazone-methyl	Xx	g ai/ha	Triazolone
MSO	1.25	%v/v	
AMS	4500	g/ha	

*Abbreviations*: *NIS* Nonionic surfactant, *AMS* Ammonium sulfate, *MSO* Methylated seed oil, *COC* Crop oil concentrate

### Dose–response study

Whole-plant dose–response studies with three putative primisulfuron-resistant shattercane biotypes and one known primisulfuron susceptible biotype were conducted in a greenhouse at the University of Nebraska-Lincoln in 2020–21. Seeds of each shattercane biotype were sown in 10 × 10 × 12 cm plastic pots containing potting mix (Metromix pottingmedia, The Scotts Company, Marysville, OH). Plants were supplied with adequate nutrients and water and were kept in the greenhouse with daytime temperature of 25 ± 2º C and a night time temperature of 18 ± 3º C, 70% − 75% relative humidity, anda 16 − h photoperiod. After emergence, seedlings were thinned to one plant per pot. Pots were arranged in a completely randomized design with six replications. A single plant per pot was considered as an experimental unit. Herbicide treatments included seven use rates of primisulfuron (0, 0.5 x, 1 x, 1.5 x, 2 x, 4 x, and 8 x), where 1.0 × was the recommended field use rate of primisulfuron (40 g ai ha − 1) and each treatment was mixed with liquid ammonium sulfate at 2.5% v/v and crop oil concentrate 0.5% v/v. Primisulfuron treatments were prepared in distilled water and applied using a single-tip chamber sprayer (DeVries Manufacturing Corp, Hollandale, MN) fitted with 8001E nozzle (Teejet, Spraying Systems Co., Wheaton, IL) calibrated to deliver 190 L ha − 1 carrier volume at 207 kPa. After primisulfuron treatment, plants were returned to the greenhouse. The experiment was repeated twice with the same procedure. Visible shattercane control was assessed 14 and 28 days after treatment (DAT) using a scale ranging from 0% (no control) to 100% (complete control). The scale of observation was from 0 to 100% with 0% being no injury and 100% being plant death. This is a standard method being used in weed science literature to evaluate the response of plant/weed to herbicides applied.. Control estimates were based on symptoms such as chlorosis, necrosis, stand loss, and stunting of the treated plants compared with nontreated control plants. Above-ground shoot biomass of each weed species was harvested from the base of the plant at 28 DAT, oven-dried for 5 d at 60º C, and dry weight was determined. Control estimates and shoot biomass reduction data (as a percentage of nontreated control) were regressed over herbicide treatments using a four-parameter log-logistic model [39].Y = C + {D-C/1 + exp [B (log X—log E)]} Where Y is the response variable (percent shattercane control or percent reduction in shoot biomass), C is the lower limit, D is the upperlimit, B is the slope of the line, E is primisulfuron rate resulting in a 50% control (known as ED50) or 90% control (known as ED90), and X is primisulfuron rate.

### DNA Extraction, Amplification, and Sequencing of the ALS Gene

The seeds from three shattercane mutants P2-205, P8-30, and P9-102, wild-type shattercane (susceptible), and sorghum genotype (CK60), a short, photoperiod-sensitive, late-maturing U.S. grown public sorghum line, were grown in pots with seven-centimeter diameternder a 16/8 h photoperiod at 25º C (day) and 18º C (night). The leaf tissues from three-week-old seedlings (four leaves stage) were collected from five plants of each biotype, frozen in liquid nitrogen, and stored at -80º C until DNA extraction. Total genomic DNA was extracted from leaf tissues of all biotypes using a modified hexadecyltrimethyl-ammonium bromide (CTAB) protocol [40]. The quality of total genomic DNA was assessed with a NanoDrop 1000 spectrophotometer with 260 and 280 nm of excitation and emission energy, respectively, and by resolution on a 1% non-denaturing agarose gel. Each DNA sample was diluted to a final concentration of 10 ng µl-1 and utilized for polymerase chain reactions (PCR). To assess a possible molecular basis for herbicide resistance, the ALS genes from three shattercane biotypes, P2-205, P8-30, and P9-102, showed resistance to primisulfuron and wild-type shattercane (susceptible) and were sequenced and compared. Sequencing primers were designed based on the corn sequence [41] (Table 6). They were located at approximately 500-bp intervals inforward and reversed orientation, providing 2- to fourfold coverage of the ALS gene. In three PCR reactions, the primers were used to amplify the ALS gene fragments from the genomic DNA of the selected mutants and the wild-type shattercane. The PCR reactions contained 40 ng of template DNA, 0.2 µM each of the forward and reverse primers, 200 µM dNTPs, 1.5 mM MgCl2, and 1.0 unit of Phusion high fidelity DNA polymerase (Thermo Scientific, #F-530S) with 1 × concentration of supplied buffer in a total volume of 25 µl. ALS gene fragments were amplified by subjecting the reactions to 4-min incubation at 95ºC, followed by 35 cycles of a denaturation step at 94º C for 45 s, an annealing stepat 55º C for 45 s, an extension step at 72º C for 1.5 min, and then a final extension step at 72º C for 5 min. To obtain the full-length sequence of ALS from shattercane, primers were created to amplify overlapping fragments of the entire gene. The experiment was repeated three times.The PCR-amplified products were resolved on a 1% (wt/v) agarose gel containing one μl ethidium bromide at 10 mg ml-1. The desired PCR fragments were excised from the gel, purified using Qiagen Gel Extraction Kit, and used for sequencing (Eurofins MWG Operon). Each PCR product was sequenced in both forward and reverse directions using ALS gene-specific primers to minimize sequencing errors. The generated nucleotide sequences from each sample were aligned using Bioedit sequence alignment editor software (Bioedit v7.1.10, Ibis Biosciences, Carlsbad, CA). The aligned sequences were compared with the sorghum genome [42] by pair-wise alignment to check the coverage of the gene by each primer. After alignment, the over lapped regions of the fragments were aligned to assemble a contiguous and complete sequence from each sample. Completely aligned sequences of the three mutants were compared with the wild-type shattercane sequence using ClustalW (ClustalW2- European Bioinformatics Institute, www.ebi.ac.uk/Tools/msa/clustalw2/) for single nucleotide substitutions. The nucleotide sequence from each mutant, wild-type shattercane, and CK60 was translated to the corresponding amino acid sequence and compared with the wild-type sequence to identify amino acid substitutions using ClustalW.

**Table 6:** Sequence of primers used to amplify the acetolactate synthase (ALS) sequence from the mutants and wild type biotypes of shattercane from Nebraska

Set	Primer	Nucleotide sequence	Amplified size (bp)
1	Forward	GTGCCCCCGCCCCAAACCCT	1–443
	Reverse	ACTAGGTTGGTGGCGCCGGG	
2	Forward	CACCTCCGGCCCCGGCGCCA	434–1071
	Reverse	CAATCTTCCCTGTCACACGA	
3	Forward	GGTTTGATGATCGTGTGACAGG	1065–1520
	Reversed	TACGCCCCAAGACCAGCTGAAGA	
4	Forward	GCAGTGGTTGTCTTCAGCTGGT	1509–1916
	Reversed	GATCATAGGCAACACATGATCCT	
5	Forward	GTGATGGCAGGACTGTGTAT	1910–2290
	Reversed	CGTACAGAACCACTGCATAG	
6	Forward	TCCGTGTGACAAAGAAGAGCGAA	1800–2280
	Reversed	GAGGCGTACAGAACCACTGCATAG	

## Conclusions

The ALS sequences and markers described in this study create tools for monitoring resistance genes in natural populations and commercial production. The goal is to develop and deploy herbicide-resistant hybrids through marker-assisted selection rapidly. Selection pressure from ALS inhibitors such as imazamox increased the risk of further development of resistant weed populations compared to herbicides with other sites of action; consequently, there are several point mutations within the ALS gene that can confer resistance to this particular class of ALS inhibitor [2, 43]. New herbicides have not been explicitly developed for grain sorghum, and glyphosate-resistant sorghum is not likely to be developed due to its possible transfer of resistance alleles into shattercane, devaluing the technology in corn and soybean. In fact, because of the high cost of developing, testing, and registering herbicides, very few new herbicides are being developed, even in major crops such as corn and soybean. Therefore, the three ALS- resistant shattercane mutants described here may expand resistance to a greater spectrum of ALS-inhibiting herbicides, allowing the development of new and more effective ALS mutations for rapid deployment in sorghum breeding programs.

## Data Availability

The datasets used and/or analyzed during the current study are available from the corresponding author on reasonable request.
